# Patency and adverse outcomes of sequential vs. individual saphenous vein grafts in coronary artery bypass: A meta-analysis

**DOI:** 10.3389/fcvm.2022.944717

**Published:** 2022-07-22

**Authors:** He Jiao, Jinghui Li, Yunpeng Bai, Zhigang Guo

**Affiliations:** ^1^Academy of Medical Engineering and Translational Medicine, Tianjin University, Tianjin, China; ^2^Department of Cardiac Surgery, Chest Hospital, Tianjin University, Tianjin, China; ^3^Clinical School of Thoracic, Tianjin Medical University, Tianjin, China

**Keywords:** coronary artery bypass graft, sequential, individual, graft failure, all-cause mortality, revascularization

## Abstract

**Objectives:**

To undertake a systematic review and meta-analysis of cohort studies to compare the patency and adverse outcomes of sequential and individual saphenous vein grafts (SVGs) in coronary artery bypass grafting (CABG).

**Methods:**

We searched PubMed, Embase, and the Cochrane Library for cohort studies. Endpoints for vein graft failure, perioperative and follow-up adverse events were extracted as risk ratio (RR) with 95% confidence intervals (95% CI). Statistical heterogeneity across the studies was examined using the I^2^ statistic. Potential of publication bias was evaluated quantitatively by the Egger's test. Sensitivity analysis was also performed to assess the robustness of our outcomes.

**Results:**

The 15 studies were analyzed, including 22,004 patients, 4,580 grafts, and seven different adverse events under individual or sequential CABG. The sequential group had inferior graft failure (RR = 0.68; 95% CI, 0.60–0.77) and long-term mortality (RR = 0.76; 95%CI, 0.61–0.95), but with an increased risk of perioperative repeat revascularization (RR = 1.58; 95%CI, 1.16–2.14) than the individual group.

**Conclusion:**

Taken together, our analysis of the aggregated evidence comparing the sequential and individual saphenous vein grafts for coronary heart disease patients showed that the use of the sequential graft was associated with inferior graft failure and long-term mortality respectively, but with an increased risk of perioperative repeat revascularization. According to our study, both surgical techniques have their own advantages in efficacy and safety, and the selection of surgical techniques should be based on patients and surgeons. Sequential saphenous vein grafts should be more recommended to experienced surgeons in order to both reduce perioperative adverse events and improve long-term patency.

**Systematic review registration:**

https://www.crd.york.ac.uk/PROSPERO/, identifier CRD42022326992.

## Introduction

Coronary heart disease (CHD) is the leading global cause of death, with 8.9 million deaths in 2019, representing 16% of all deaths (1). Coronary artery bypass grafting (CABG) is one of the common therapies for CHD, especially preferred for more complex cases affecting multiple vessels or the left main coronary artery. Generally, the most commonly performed approach in CABG is connecting the left internal mammary artery (LIMA) to the left anterior descending artery (LAD), combined with one or more saphenous vein grafts (SVGs) (2, 3). SVGs can be performed as an individual graft with one distal anastomosis or as a sequential graft with two or more distal anastomoses.

In previously published literature, the optimal SVG strategy remains controversial. Sequential grafting has been claimed to offer theoretical advantages, including higher graft flow, greater conservation of conduit and reduced aortic manipulation (4–6). The advocates of sequential SVGs have found superior mid-term patency for sequential grafts and anastomoses (7, 8). However, some researchers have argued that sequential grafting is a more challenging and adventurous technique because of the reliance of multiple distal anastomoses on a shared proximal inflow (9, 10). A proximal occlusion could be fatal as a larger section of the myocardium is at risk of ischemia (11).

In addition, many studies comparing individual SVG to sequential SVG in CHD patients were performed 30 years ago and are no longer applicable to the current level of CABG surgery (2, 4, 7). Currently, there are few large contemporary studies comparing individual vein grafts to sequential vein grafts in CABG. Our objective was to undertake a systematic review and meta-analysis of cohort studies to compare the patency and adverse outcomes of sequential and individual SVGs in CABG.

## Materials and methods

The protocol for this systematic review (CRD42022326992) was conducted in accordance with the Preferred Reporting Items for Systematic Reviews and Meta-Analysis statement (PRISMA) (12).

### Search strategy

**Figure 1:**
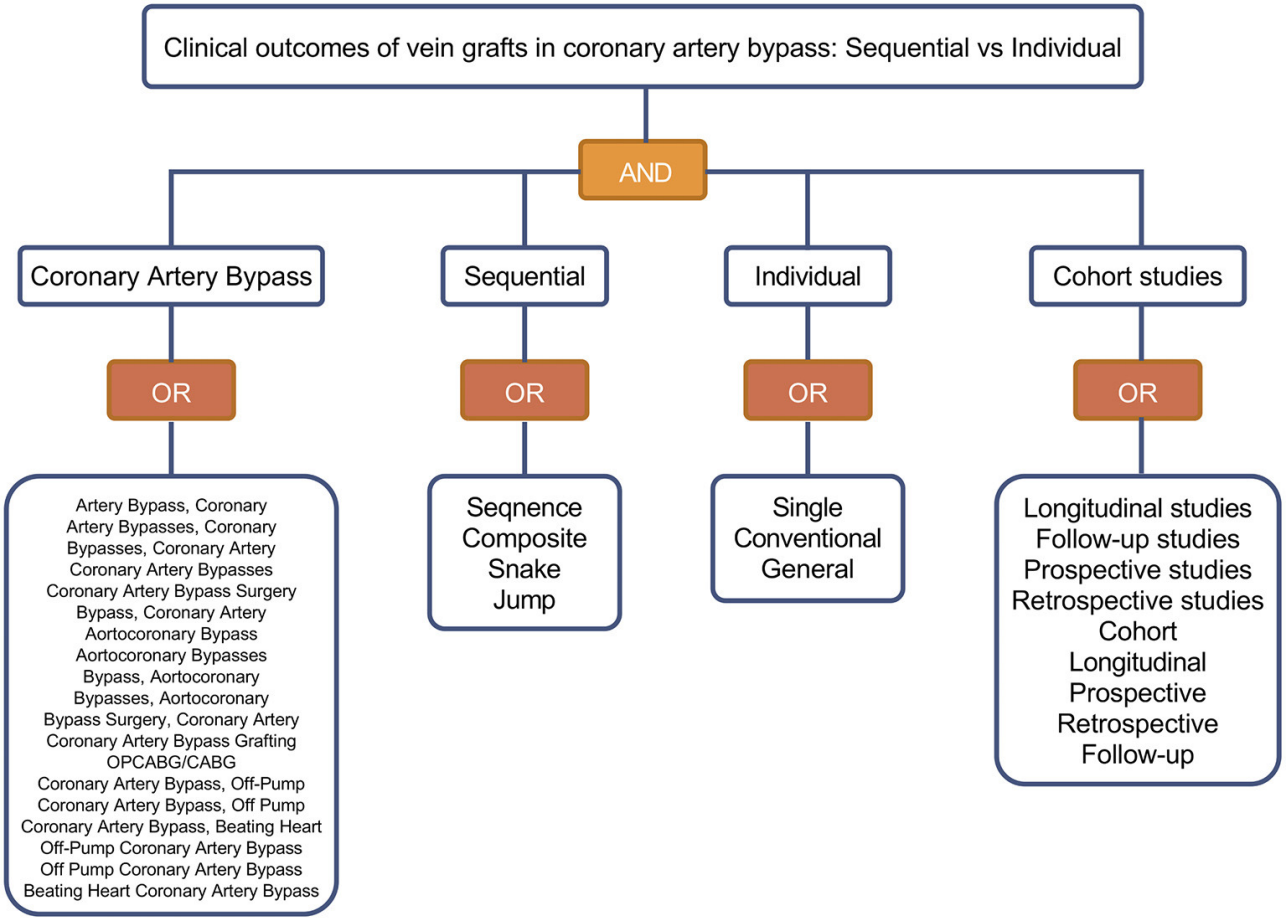
MeSH search terms used when searching databases.

We carried out a systematic literature search to identify relevant studies in PubMed, Excerpta Medica Database (EmBase), and the Cochrane Library. The search included studies from January 2000 to March 2022. The following primary medical subject headings (MeSH) and free terms were used in the structured search strategy: “coronary artery bypass,” “sequential,” “individual” and “cohort studies” (Figure 1). The search strategies were adjusted appropriately to meet the requirements of the different databases and the full search strategies for these databases are available in Supplementary Tables S1–S3. The search was also restricted to the English language and studies on humans. In addition to database search, recent meta-analyses and reviews on this topic were hand-searched for potential additional literature. And the reference lists of the retrieved literature were examined to exclude duplicate reports in the same cohort.

### Inclusion and exclusion criteria

The included studies satisfied all of the following criteria: (1) CHD patients undergoing isolated CABG; (2) Trials published publicly in databases and/or on the Internet; (3) Cohort studies comparing sequential SVG (not including Y-bridge and T-bridge) to individual SVG; (4) Related clinical outcomes reported, such as saphenous vein graft failure, all-cause mortality, non-fatal myocardial infarction (MI), stroke and repeat revascularization in different terms.

Studies were excluded if they did not report the related clinical outcomes or were reported only as abstracts. In addition, we also excluded reviews, meta-analyses, animal research, and case reports. When necessary, corresponding authors were contacted to request the original data.

### Outcomes

The short-term outcomes for this meta-analysis were perioperative (30 days or in-hospital) adverse outcomes after CABG, including perioperative all-cause mortality, stroke, MI and repeat revascularization. Repeat revascularization was defined as a postoperative percutaneous coronary intervention or repeated CABG (13). The follow-up outcomes in our meta-analysis were saphenous vein graft failure and mid- or long-term adverse outcomes, including all-cause mortality and repeat revascularization. Vein graft failure was defined as a ≥50% stenosis or occlusion at follow-up angiography or CT-angiography (14).

### Data extraction

Two independent reviewers assessed the studies identified from databases to follow all eligibility criteria. Information was extracted from each included study on:

(1) Descriptive characteristics of study (first author, published year, setting);
(2) Sample characteristics (average age, female percentage, other matching features, and surgical methods);
(3) Follow-up of study (sample size, follow-up time, and follow-up rate);
(4) Clinical outcomes (saphenous vein graft patency, all-cause mortality, MI, stroke and repeat revascularization in different terms).

### Quality assessment

Following the Newcastle-Ottawa Scale (NOS) (15), we considered eight items to evaluate the quality of the included studies: (1) Adequate definition of cases; (2) Representativeness of the cases; (3) Selection of controls; (4) Definition of controls; (5) Control for important factor; (6) Ascertainment of exposure; (7) Same method of ascertainment for cases and controls; (8) Nonresponse rate. The NOS score ranges from zero to nine points. Low-quality research was defined as five points or below, and high-quality literature was defined as eight or above.

### Statistical analysis

The statistical analysis was performed using RevMan 5.4 (Nordic Cochrane Center, Collaboration) and Stata 14.0 (Stata Corp, College Station, Texas, USA) software. The study effects were measured using risk ratio (RR) as the pooled estimate, and the results were analyzed based on 95% confidence intervals (CIs). Two-sided *p*-values <0.05 were considered statistically significant (16). Statistical heterogeneity across the studies was examined using the I^2^ statistic. Boundaries of <25, 25–50 and >50% were used to define low, moderate and high levels of heterogeneity, respectively (17). If no substantial heterogeneity (I^2^ ≤ 50%) was noted, a fixed-effect model was used to pool the results. If substantial heterogeneity was (I^2^ > 50%) observed, a random-effect model was used for statistical analysis. Potential of publication bias was evaluated visually by funnel plot and quantitatively by the Egger's test. Sensitivity analysis was also performed to assess the robustness of our outcomes.

## Results

### Study selection

**Figure 2:**
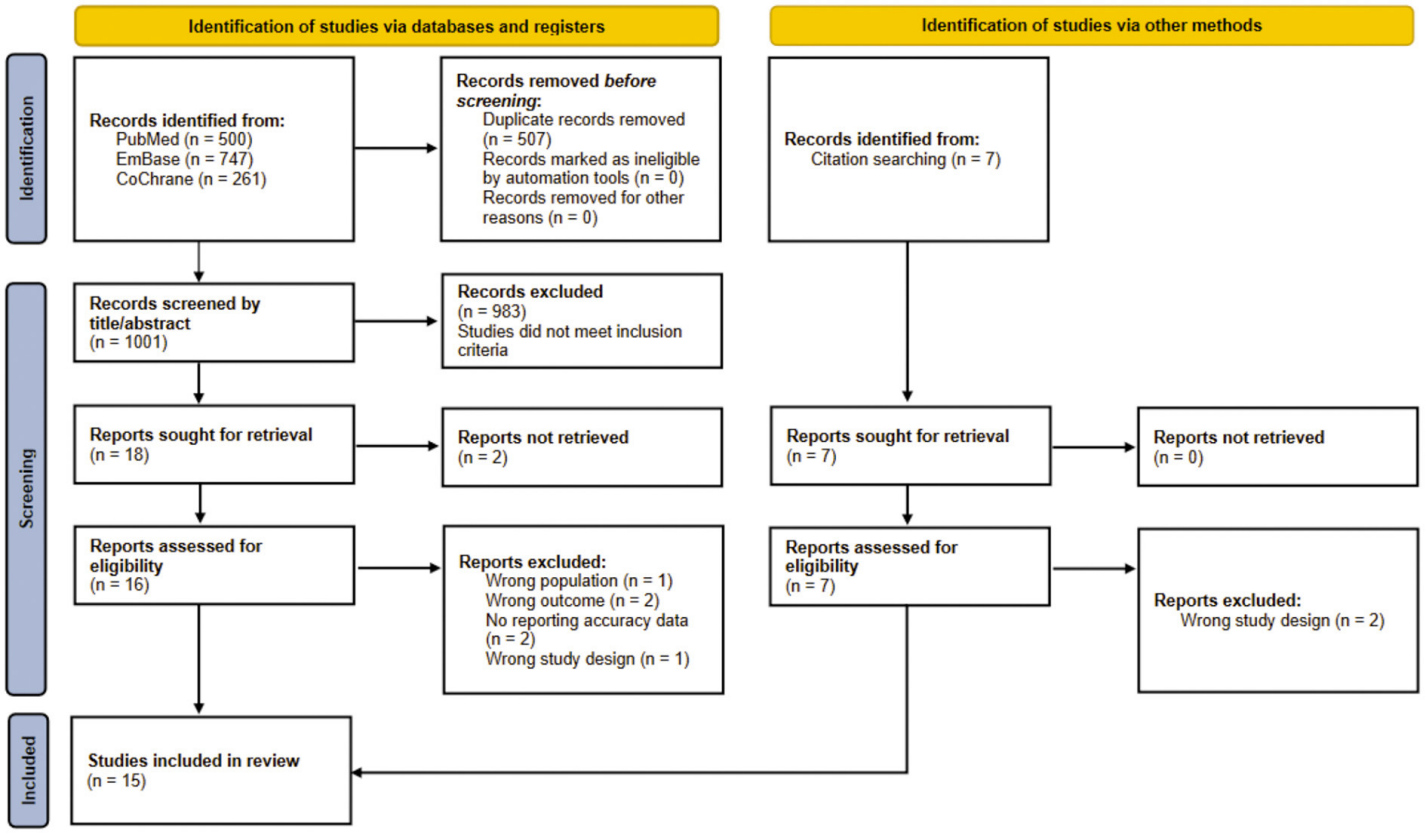
Study selection flow diagram.

A total of 1,515 studies were identified through a preliminary database search, including 500 articles from PubMed, 747 articles from Embase, 261 articles from Cochrane, and seven related articles obtained through reference lists of the retrieved literature. 1Thousand hundred and one non-duplicate citations for titles and abstracts screening were found. Twenty-three relevant studies were checked for full-text assessment. Of these, a total of 15 studies were included in this review. The detailed process of inclusion in this meta-analysis is shown in Figure 2.

### Study characteristics

Of the included 15 studies, nine were published since 2010 and six were in the 2010s, respectively conducted in 10 countries (China, South Korea, Sweden, Denmark, Japan, Canada, Brazil, Italy, Turkey, and Belgium). Altogether, 22,004 participants were included from 15 clinical trials with a weighted mean age of 66 years and a predominance of male patients. Of the selected studies, four studies included patients performed on-pump CABG only and two contained patients conducted off-pump CABG only.

The sample sizes of most studies were <500. Only two large contemporary studies (N > 6,000) were included. The selected studies were with a mean follow-up length ranging from 12 to 115.2 months. The majority of studies had a follow-up rate of more than 80% and four articles were not described.

Of the included studies, nine studies reported the patency of the saphenous vein grafts and six articles described the adverse events after CABG. Perioperative (30 days or in-hospital) mortality was reported in six studies, in-hospital stroke was in four studies, and perioperative (30 days or in-hospital) repeat revascularization in three studies. For follow-up outcomes, all-cause mortality was reported in six studies and repeat revascularization was described in four studies respectively. The characteristics of the included studies are displayed in Supplementary Table S4.

### Study quality evaluation

The quality of the included studies was evaluated according to the NOS scale, and the results are shown in Table 1. All 15 studies were medium-to-high quality cohort studies, and the scores ranged from six to nine points.

**Table 1:** Quality of the included studies.

**No.**	**References**	**Setting**	**The quality of include studies**			
			**Selection**	**Comparability**	**Exposure**	**Score**
NO.1	Zeng et al. (18)	China	4	0	2	6
NO.2	Park et al. (19)	South Korea	4	2	3	9
NO.3	Wallgren et al. (13)	Sweden	4	2	3	9
NO.4	Skov et al. (20)	Denmark	4	2	3	9
NO.5	Takazawa et al. (21)	Japan	4	0	2	6
NO.6	Xiao et al. (22)	China	4	2	3	9
NO.7	Kim et al. (23)	South Korea	4	2	3	9
NO.8	Ouzounian et al. (24)	Canada	4	2	3	9
NO.9	Gao et al. (25)	China	4	0	2	6
NO.10	Silva et al. (26)	Brazil	4	0	2	6
NO.11	Onorati et al. (27)	Italy	4	2	2	8
NO.12	Farsak et al. (28)	Turkey	4	2	2	8
NO.13	Souza et al. (29)	Sweden	4	0	3	7
NO.14	Vural et al. (5)	Turkey	4	2	2	8
NO.15	Dion et al. (6)	Belgium	4	0	2	6

### Main meta-analysis

#### Graft failure

**Figure 3:**
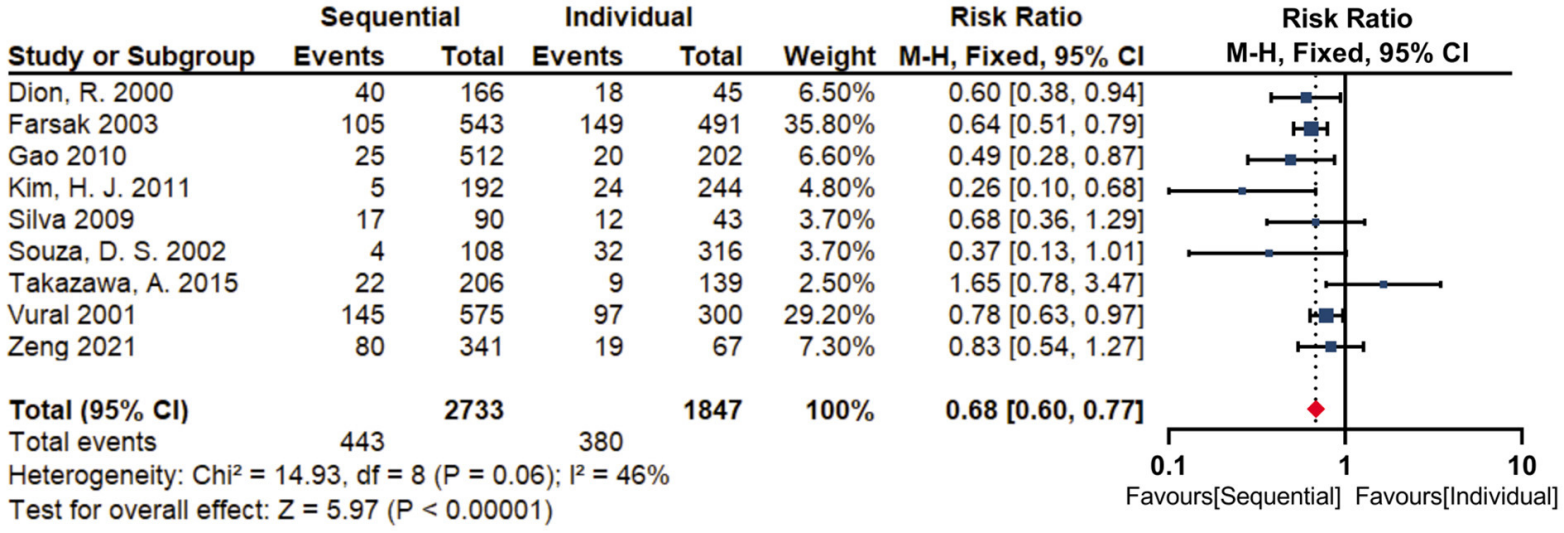
Forest plot for graft failure. Nine studies reported the failure of the saphenous vein grafts, revealing that sequential group (16.2%) was associated with a significantly lower risk of graft failure (RR = 0.68; 95% CI, 0.60–0.77, *P* < 0.001) compared with individual group (20.6%).

**Figure 4:**
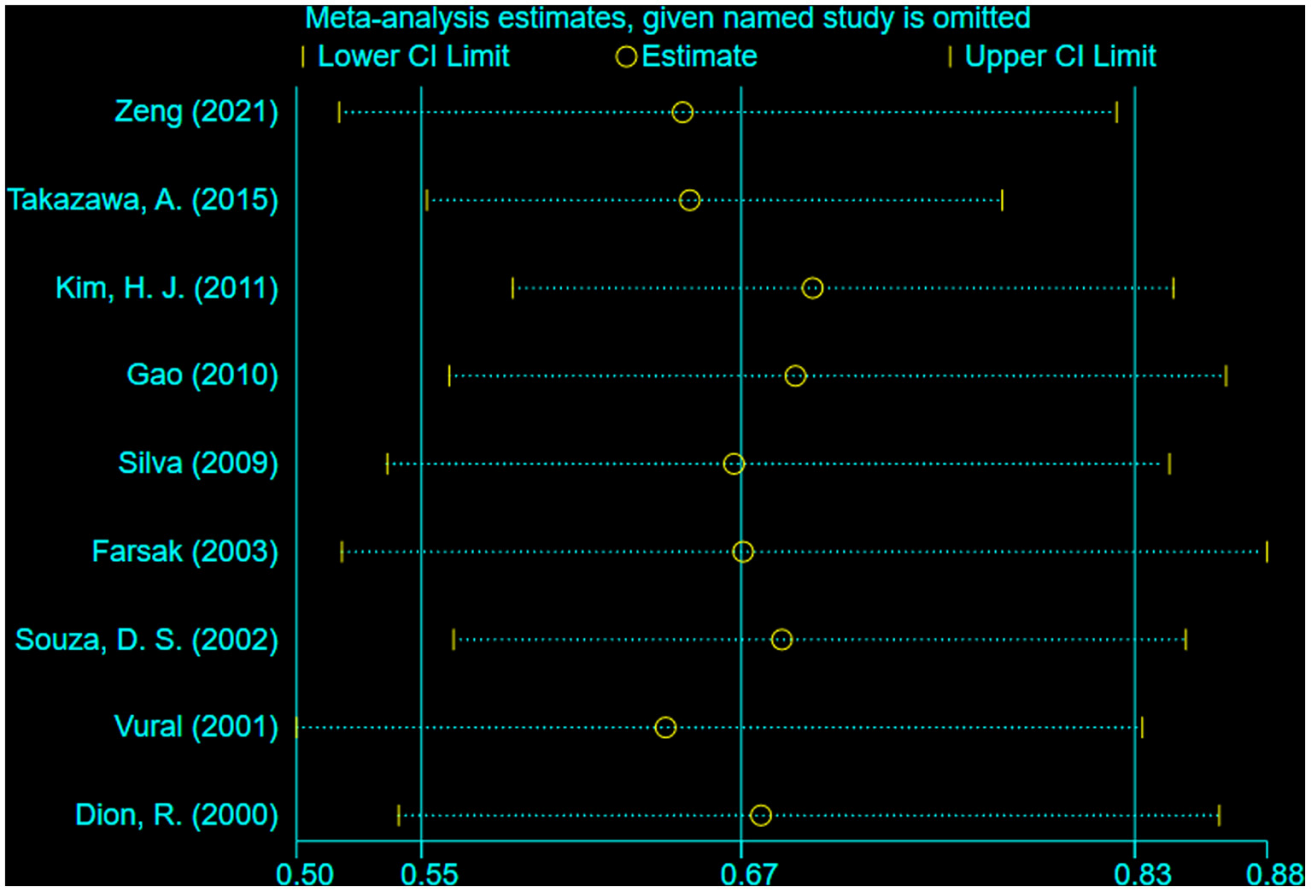
Sensitivity analysis for graft failure (Leave-one-out analysis). By sequentially eliminating each study from nine studies reporting graft failure, sensitivity analysis showed that no particular study could largely influence the result.

Nine studies reported the failure of the saphenous vein grafts, revealing that sequential group (16.2%) was associated with a significantly lower risk of graft failure (RR = 0.68; 95% CI, 0.60–0.77, *P* < 0.001) compared with individual group (20.6%) (Figure 3). There was moderate statistical heterogeneity between the included studies (I^2^ = 46%, *P* = 0.06). By sequentially eliminating each study from the data pool, sensitivity analysis showed that no particular study could largely influence the result (Figure 4). There was no significant publication bias when examined by the Egger test (*P* = 0.489).

Considering the significant inconsistencies in evaluation method, failure definition, follow-up rate, surgery method and follow-up time, subgroup analyses were conducted to exclude these possible confounding factors.

Eight studies reported on the evaluation methods of grafts, of which two were examined by CT and six were assessed by angiography (Supplementary Figure S1A). Subgroup analyses found that there was high statistical heterogeneity between CT subgroup and angiography subgroup in our meta-analysis results (I^2^ = 78.1%, *P* =0.03). In CT subgroup with a low statistical heterogeneity (I^2^ = 22%, *P* =0.26), graft failure rate was 4.3% in sequential group vs. 9.7% in individual group (RR = 0.4; 95% CI, 0.24–0.65, *P* = 0.0002). In angiography subgroup with no statistical heterogeneity (I^2^ = 0%, *P* = 0.51), graft failure rate was 21.4% in sequential group vs. 25.9% in individual group (RR = 0.69; 95% CI, 0.60–0.79, *P* < 0.001). Subgroup analyses based on graft evaluation found there may be no difference in risk of graft failure.

Nine studies clearly defined grafting failure, of which five studies defined the degree of occlusion as 100%, and four studies defined the diameter of graft stenosis at ≥50% (Supplementary Figure S1B). Subgroup analyses found that there was moderate statistical heterogeneity between occlusion subgroup and stenosis subgroup (I^2^ = 39%, *P* = 0.2), and revealed that lower vein graft failure rate in sequential group compared with individual group in occlusion subgroup (RR = 0.65; 95% CI, 0.57–0.75, *P* <0.001) but not in stenosis subgroup (RR = 0.81; 95% CI, 0.60–1.10, *P* = 0.018) respectively.

Five studies reported on the follow-up rate, of which three studies featured follow-up rates of ≥80%, while two studies had follow-up rates of <80% (Supplementary Figure S1C). Subgroup analyses found that there was high statistical heterogeneity between subgroup with follow-up rate of ≥80% and subgroup with follow-up rate of <80% in our meta-analysis results (I^2^ = 57.8%, *P* = 0.12). In high-follow-up-rate subgroup with a moderate statistical heterogeneity (I^2^ = 35%, *P* = 0.21), graft failure rate was 6.7% in sequential group vs. 11.3% in individual group (RR = 0.42; 95% CI, 0.26–0.68, *P* = 0.0004). In low-follow-up-rate subgroup with no statistical heterogeneity (I^2^ = 0%, *P* = 0.82), graft failure rate was 20.5% in sequential group vs. 31.2% in individual group (RR = 0.63; 95% CI, 0.52–0.77, *P* < 0.001). Subgroup analyses based on follow-up rates found there may be no difference in risk of graft failure.

Four studies reported on different surgery methods, of which two studies featured off-pump CABG, while two studies used on-pump CABG respectively (Supplementary Figure S1D). Subgroup analyses found that there was no statistical heterogeneity between on-pump CABG and off-pump CABG in our meta-analysis results (I^2^ = 0%, *P* = 0.36). In on-pump CABG subgroup with no statistical heterogeneity (I^2^ = 0%, *P* = 0.34), graft failure rate was 16.1% in sequential group vs. 13.9% in individual group (RR, 0.52; 95% CI, 0.33–0.80, *P* = 0.003). In off-pump CABG subgroup with a high statistical heterogeneity (I^2^ = 52%, *P* = 0.15), graft failure rate was 12.3% in sequential group vs. 14.5% in individual group (RR = 0.67; 95% CI, 0.48–0.94, *P* =0.02). Subgroup analyses based on surgical methods found there may be no difference in risk of graft failure.

Seven studies reported on the follow-up duration, of which five studies featured follow-up time of ≥5 years, while two studies had follow-up time of <5 years (Supplementary Figure S1E). Subgroup analyses found that there was a moderate statistical heterogeneity between subgroup with follow-up time of ≥5 years and subgroup with follow-up time of <5 years in our meta-analysis results (I^2^ = 32.3%, *P* = 0.22). In ≥5 years subgroup with a high statistical heterogeneity (I^2^ = 59%, *P* = 0.03), graft failure rate was 15.1% in sequential group vs. 21.9% in individual group (RR = 0.69; 95% CI, 0.60–0.79, *P* < 0.001). In <5 years subgroup with no statistical heterogeneity (I^2^ = 0%, *P* = 0.34), graft failure rate was 16.1% in sequential group vs. 13.9% in individual group (RR = 0.52; 95% CI, 0.33–0.80, *P* = 0.003). Subgroup analyses based on different follow-up time showed there may be no difference in risk of graft failure.

#### Perioperative adverse events

Six studies reported perioperative all-cause mortality, with a high statistical heterogeneity between the included studies (I^2^ = 60%, *P* = 0.04). A random-effect model was used for statistical analysis. Our meta-analysis revealed that sequential group (2.1%) was without a significant risk of perioperative mortality (RR = 1.2; 95% CI, 0.79–1.81, *P* = 0.39) compared with individual group (1.6%) (Supplementary Figure S2A). By sequentially eliminating each study from the data pool, sensitivity analysis showed that no particular study could largely influence the result (Supplementary Figure S3). There was no significant publication bias when examined by the Egger test (*P* = 0.652).

Four studies reported in-hospital stroke, revealing that no risk difference (RR = 1.03; 95% CI, 0.77–1.37, *P* = 0.85) between sequential group (2.0%) and individual group (2.0%) (Supplementary Figure S2B). There was no statistical heterogeneity between the included studies (I^2^ = 0%, *P* = 0.9). A stable meta-analysis outcome was obtained by excluding selected studies one by one. No significant evidence of publication bias was found using the Egger's test in this endpoint.

**Figure 5:**
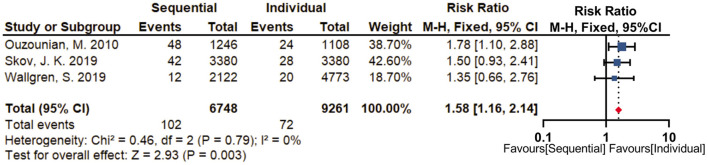
Forest plot for perioperative repeat revascularization. Three studies reported perioperative repeat revascularization, revealing that sequential group (1.5%) was associated with a significantly higher risk of perioperative repeat revascularization (RR = 1.58; 95% CI, 1.16–2.14, *P* = 0.003) compared with individual group (0.7%).

Three studies reported perioperative repeat revascularization, revealing that sequential group (1.5%) was associated with a significantly higher risk of perioperative repeat revascularization (RR = 1.58; 95% CI, 1.16–2.14, *P* = 0.003) compared with individual group (0.7%) (Figure 5). There was no statistical heterogeneity between the included studies (I^2^ = 0%, *P* = 0.79). The limited number of selected studies did not permit sensitivity analysis and assessment of publication bias.

Four studies reported perioperative myocardial infarction, of which one study (13) missed in-hospital MI outcomes and other studies lacked a uniform definition of MI. Therefore, perioperative MI outcome was not analyzed in our meta-analysis.

#### Mid-term adverse events

There was no significant publication bias when examined by the Egger test (*P* = 0.652). Three studies reporting mid-term mortality (1~5 years) were noted (Supplementary Figure S4A), the results of which showed no statistically significant difference (RR = 0.96; 95%CI, 0.86–1.06) between sequential group (10.2%) and individual group (9.8%). There was no statistical heterogeneity among the selected studies (I^2^ = 0%, *p* = 0.51). The limited number of selected studies did not permit sensitivity analysis and assessment of publication bias.

Three studies reporting mid-term repeat revascularization rates (1~5 years) were noted (Supplementary Figure S4B), the results of which showed no statistically significant difference (RR = 1.00; 95%CI, 0.86–1.17) between sequential group (4.7%) and individual group (4.7%). There was no statistical heterogeneity among the selected studies (I^2^ = 0%, *p* = 0.49). The limited number of selected studies did not permit sensitivity analysis and assessment of publication bias.

#### Long-term adverse events

**Figure 6:**
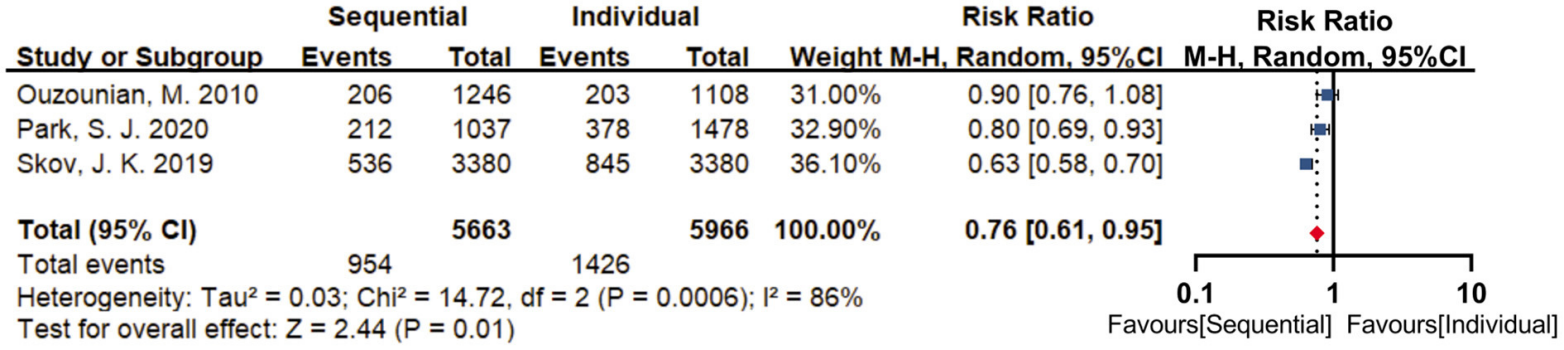
Forest plot for long-term mortality. Three studies reported long-term mortality (>5 years), with a high statistical heterogeneity between the included studies (I^2^ = 86%, *P* = 0.0006). By using a random-effect model, results revealed that sequential group (16.8%) was with a lower risk of long-term mortality (RR = 0.76; 95% CI, 0.61–0.95, *P* = 0.01) compared with individual group (23.9%).

Three studies reported long-term mortality (more than 5 years), with a high statistical heterogeneity between the included studies (I^2^ = 86%, *P* = 0.0006). A random-effect model was used for statistical analysis. Our meta-analysis revealed that sequential group (16.8%) was with a lower risk of long-term mortality (RR = 0.76; 95% CI, 0.61–0.95, *P* = 0.01) compared with individual group (23.9%) (Figure 6). The limited number of selected studies did not permit sensitivity analysis and assessment of publication bias.

## Discussion

Since Flemma et al. originally described sequential SVGs in 1971 (30), the sequential bypass technique has been widely applied in CABG surgery. Subsequently, this technology was introduced and described in detail by Bartley and his colleagues (31). However, previous literature concerning the safety and efficacy of sequential grafting have been conflicting (10, 32). Furthermore, the existing research maybe no longer reflects contemporary surgical management (33, 34). Hence, our meta-analysis was undertaken to compare patency and adverse outcomes of sequential and individual saphenous vein grafts used in CHD patients under CABG.

Sequential grafting is a technique where more than one distal anastomosis is performed to a single proximal target conduit. Theoretically, this technique can increase the hemodynamic advantage of total graft flow by improving distal runoff and thereby increase graft patency rates. According to Gao and his colleagues, compared with individual bypass grafts (*N* = 202), sequential grafts (*N* = 512) were associated with higher mean flows and superior mid- and long-term patency (25). Park et al. also reported that the sequential grafts had better patency for both before (*P* = 0.015) and after adjustment (HR = 0.61; 95% CI, 0.45 to 0.82; *P* < 0.001) by comparing 1,037 patients with sequential SVGs and 1,478 patients with individual SVGs (19). However, some studies are not consistent with the above views. Meurala et al. found that the distal segments of sequential group had a significantly lower mean graft flow compared with the individual group (32). Takazawa. et al. reported that sequential SVGs had significantly higher failure than single SVGs after an average follow-up of 14.7 months (21). Nevertheless, in this systematic review, one of our findings is that the sequential saphenous vein grafts results in superior patency than individual grafts, consistent with the first view. The more favorable outcomes of sequential bypass grafting may be attributable to hemodynamic enhancement, along with improved accuracy of intraoperative blood flow technology. Kim et al. reported that the proximal part of the sequential SVG flowed faster than the individual SVG (23). Other studies also found that sequential grafting was associated with superior mean blood flow and favorable pulsatility index (22). To some extent, these trials reflect the advantage of sequential grafts in patency.

Another finding of our study is that the sequential saphenous vein grafts are associated with lower long-term mortality, but with a significantly higher risk of perioperative repeat revascularization. As we know, sequential grafting is considered to be a more challenging revascularization technique. Just as some surgeons worried, sequential grafting typically puts “all the eggs in one basket,” that is, all distal anastomoses only rely on the same proximal graft. Occlusion of the proximal graft leads to decreased blood flow of the distal anastomoses, most likely reflected clinically in the immediate, such as re-angiography, extensive myocardial ischemia and so on. This seems to explain the high rate of perioperative repeat revascularization in the sequential group. Wallgren and colleagues found a significantly higher rate of re-angiography in the days immediately (13). According to Skov et al., sequential group were observed to have more early myocardial ischemia (20). One of our findings is similar to the previous studies. Hence, sequential techniques should be more recommended to experienced surgeons in order to reduce risk of perioperative repeat revascularization. In addition, it has been suggested that the patent distal end of the sequential graft plays a supporting role in the collateral circulation when the proximal segment is occluded (25). Therefore, a proximal occlusion of sequential graft may be not fatal in most instances. This seems to explain our results that the long-term mortality of sequential graft is better than that of individual graft. And this also suggests why sequential grafts are associated with lower long-term mortality, but with a significantly higher risk of perioperative repeat revascularization. It is possible that the collateral circulation is not established in the short-term and does not play a protective role in time. So sequential saphenous vein grafts should be recommended to experienced surgeons to both reduce perioperative adverse events and improve long-term patency.

The results of this meta-analysis must be interpreted in the context of some important limitations. Firstly, the included studies were retrospective cohort studies, which may be biased by treatment bias and confounding factors. There were differences in lesions of coronary artery in patients, surgical technique requirements, and surgeon preference, which led to possible selection bias in our study. For the above possible bias, subgroup analyses were used to evaluate the variation between sequential verse individual group. Besides, there were 6 studies with a cohort comparability score of 0 in meta-analysis and could not find differences between baseline characteristics, which may affect the estimates of the study. For this reason, we compared the results of adjusted and matched studies with those of unmatched studies. It was somewhat reassuring that the results of the two groups were similar. In addition, four studies did not report follow-up rates and the two studies was with follow-up rate of <80% in this meta-analysis. These studies raised the possibility of biased results. Finally, the analyses on myocardial infarction and long-term repeat revascularization included only few studies and were very likely underpowered.

## Conclusion

Taken together, our analysis of the aggregated evidence comparing the sequential and individual saphenous vein grafts for coronary heart disease patients showed that the use of the sequential graft was associated with inferior graft failure and long-term mortality respectively, but with an increased risk of perioperative repeat revascularization.

According to our study, both surgical techniques have their own advantages in efficacy and safety, and the selection of surgical techniques should be based on patients and surgeons. Sequential saphenous vein grafts should be more recommended to experienced surgeons in order to both reduce perioperative adverse events and improve long-term patency.

## Data availability statement

The original contributions presented in the study are included in the article/supplementary material, further inquiries can be directed to the corresponding author/s.

## Author contributions

ZG contributed to the conception and design, supervision of the study, final approval of the article and obtaining funding. YB contributed to the conception and design, literature search, data collection, analysis and interpretation and obtaining funding. HJ performed literature search, collected relevant data, carried out statistical analysis, and wrote the original manuscript. JL collected relevant data, carried out statistical analysis, polished the draft, and provided technical or logistic support. All authors contributed to the article and approved the submitted version.

## Funding

The study was supported by the Natural Fund Project of Tianjin Science and Technology Bureau (20JCZDJC00810) and Scientific Research Project of Integrated Traditional Chinese and Western Medicine of Tianjin Health Commission (2021204).

## Conflict of interest

The authors declare that the research was conducted in the absence of any commercial or financial relationships that could be construed as a potential conflict of interest.

## Publisher's note

All claims expressed in this article are solely those of the authors and do not necessarily represent those of their affiliated organizations, or those of the publisher, the editors and the reviewers. Any product that may be evaluated in this article, or claim that may be made by its manufacturer, is not guaranteed or endorsed by the publisher.
