# Supplementary figures and images for: Patency and adverse outcomes of sequential vs. individual saphenous vein grafts in coronary artery bypass: A meta-analysis
Source: Front Cardiovasc Med. 2022 Jul 22;9:944717. doi: 10.3389/fcvm.2022.944717 (PMC9355302; doi:10.3389/fcvm.2022.944717)

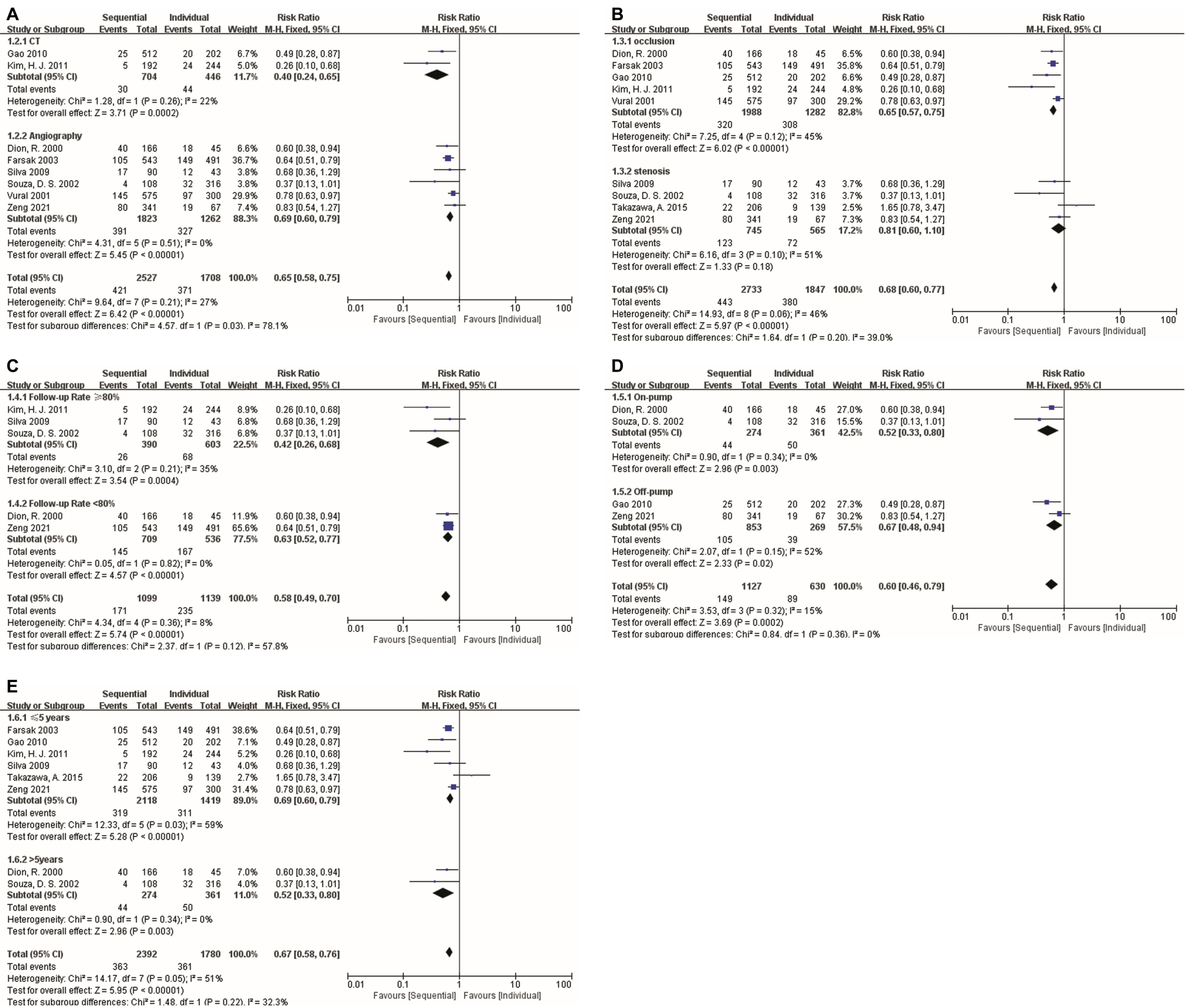

Supplement: Supplementary file 1 [file Data_Sheet_1.ZIP › Supplementary Figures/Figure S1.tif]

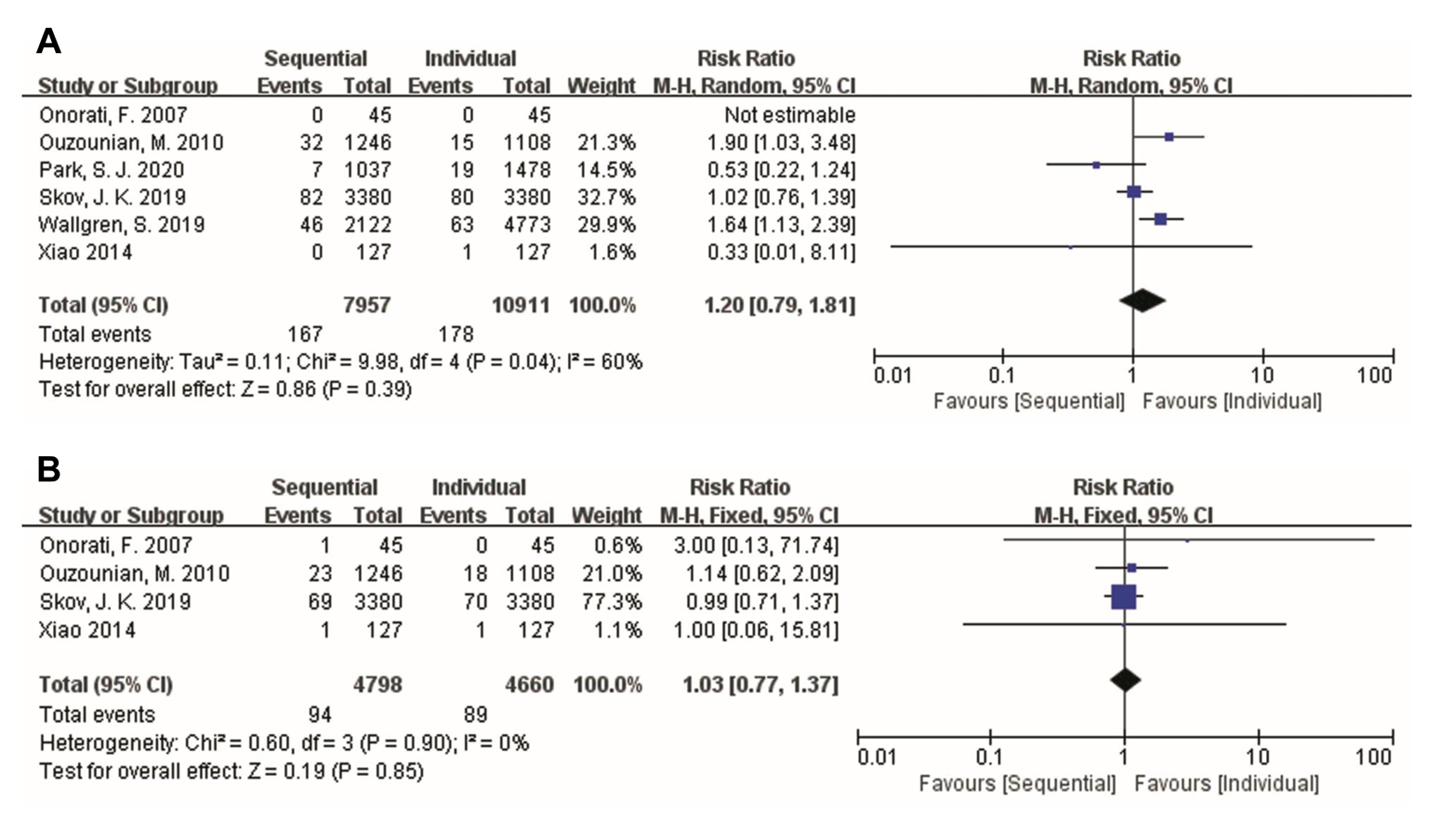

Supplement: Supplementary file 1 [file Data_Sheet_1.ZIP › Supplementary Figures/Figure S2.tif]

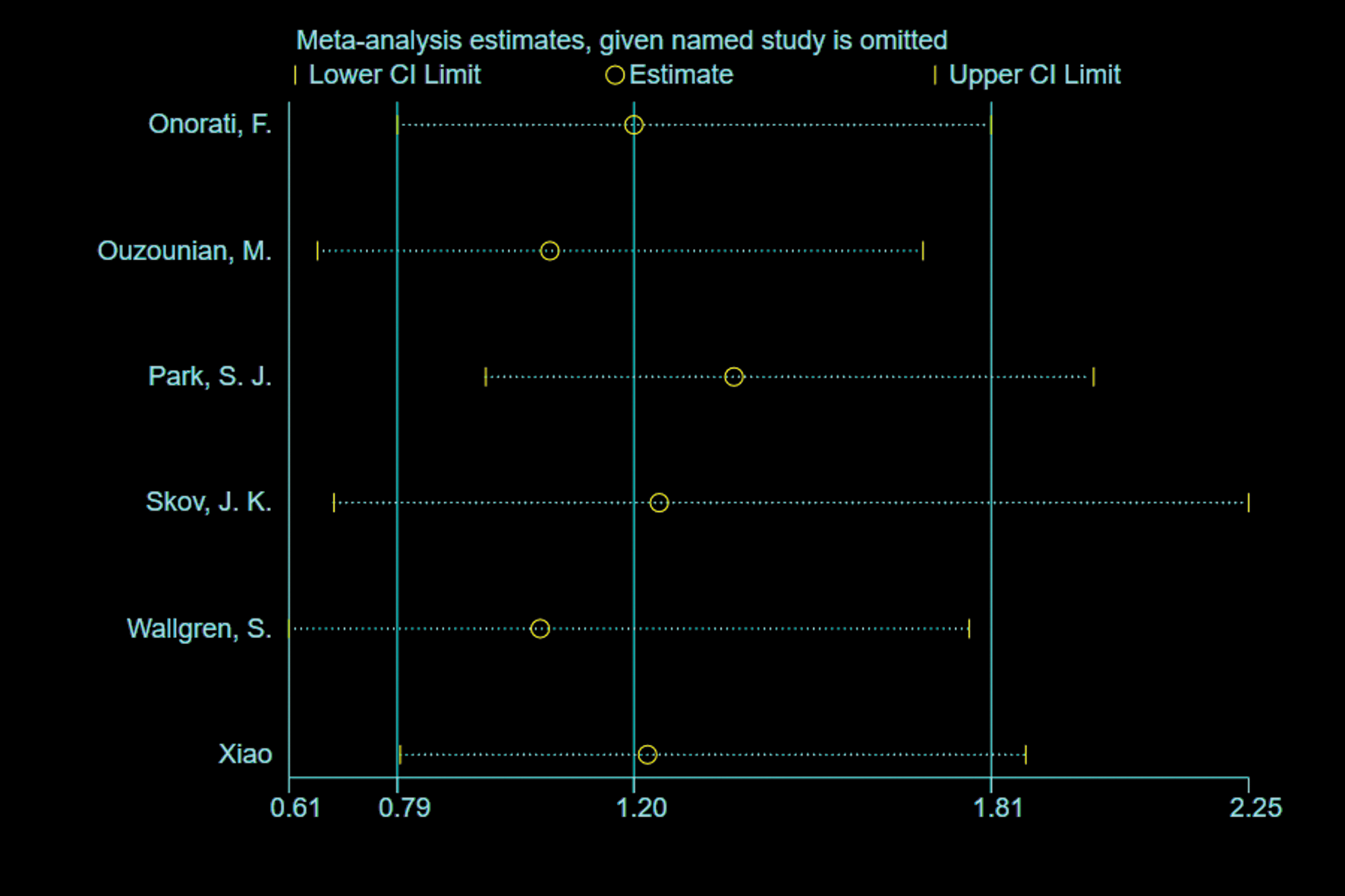

Supplement: Supplementary file 1 [file Data_Sheet_1.ZIP › Supplementary Figures/Figure S3.tif]

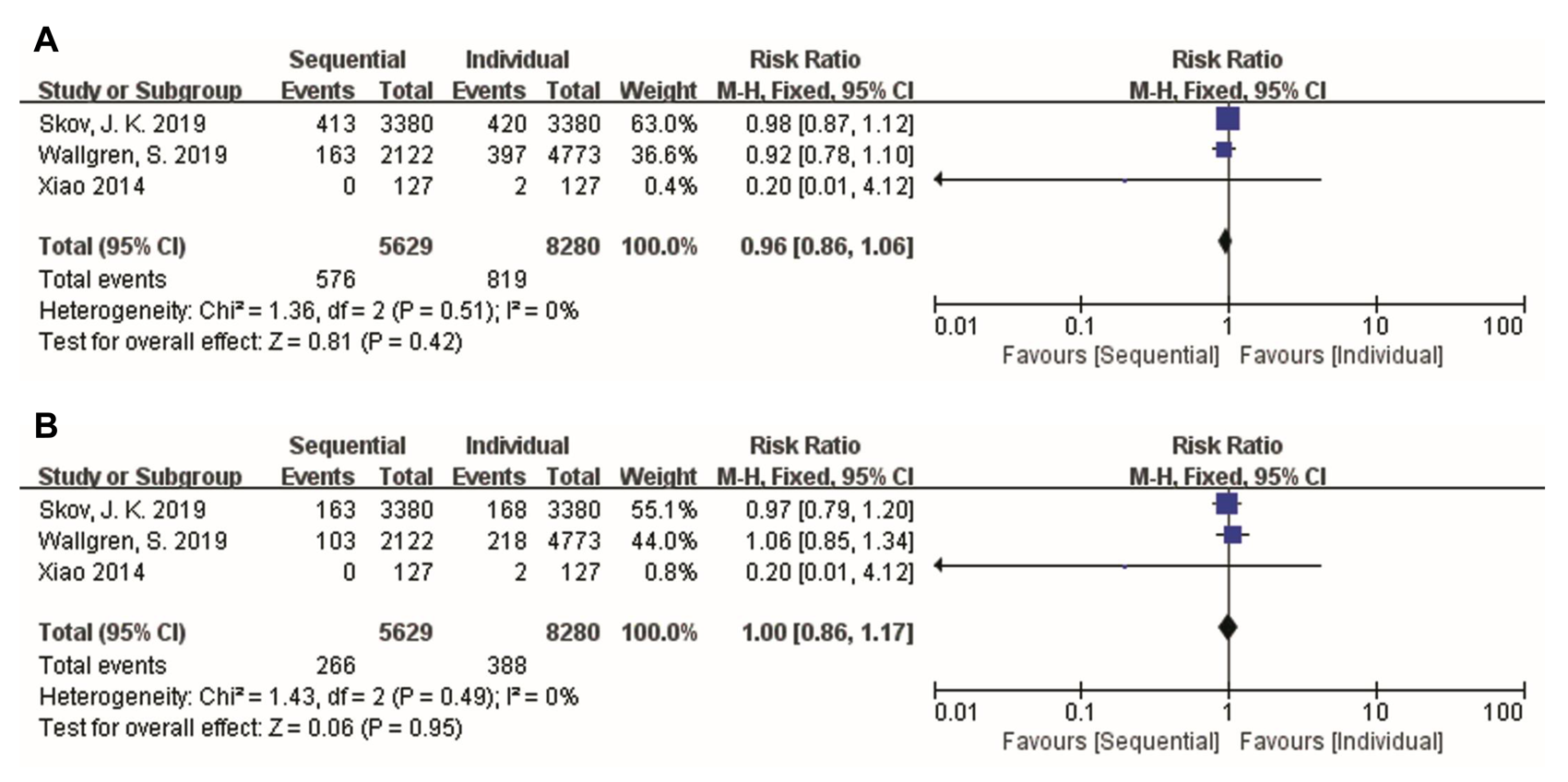

Supplement: Supplementary file 1 [file Data_Sheet_1.ZIP › Supplementary Figures/Figure S4.tif]
