# Supplementary material for: Patency and adverse outcomes of sequential vs. individual saphenous vein grafts in coronary artery bypass: A meta-analysis
Source: Front Cardiovasc Med. 2022 Jul 22;9:944717. doi: 10.3389/fcvm.2022.944717 (PMC9355302; doi:10.3389/fcvm.2022.944717)
Supplement: Supplementary file 2 [file Data_Sheet_2.docx]

Supplementary Material

# Supplementary Figures and Tables

## Supplementary Tables

**Table S1.** Full search strategy for EmBase (Searched on 03/03/2022).

| **Search number** | **Query** | **Results** |
| --- | --- | --- |
| 1 | 'coronary artery bypass graft'/exp | 80,629 |
| 2 | 'Artery Bypass, Coronary':ab,ti or 'Artery Bypasses, Coronary':ab,ti or 'Bypasses, Coronary Artery':ab,ti or 'Coronary Artery Bypasses':ab,ti or 'Coronary Artery Bypass Surgery':ab,ti or 'Bypass, Coronary Artery':ab,ti or 'Aortocoronary Bypass':ab,ti or 'Aortocoronary Bypasses':ab,ti or 'Bypass, Aortocoronary':ab,ti or 'Bypasses, Aortocoronary':ab,ti or 'Bypass Surgery, Coronary Artery':ab,ti or 'Coronary Artery Bypass Grafting':ab,ti or 'Coronary Artery Bypass':ab,ti | 61,207 |
| 3 | 'off pump coronary surgery'/exp | 6,196 |
| 4 | 'Coronary Artery Bypass, Off-Pump':ab,ti or 'Coronary Artery Bypass, Off Pump':ab,ti or 'Coronary Artery Bypass, Beating Heart':ab,ti or 'Off-Pump Coronary Artery Bypass':ab,ti or 'Off-Pump Coronary Artery Bypass':ab,ti or 'Beating Heart Coronary Artery Bypass':ab,ti or 'CABG':ab,ti or 'OPCABG':ab,ti | 38,332 |
| 5 | 'sequential':ab,ti OR 'sequence':ab,ti OR 'composite':ab,ti OR 'snake':ab,ti OR 'jump':ab,ti | 1,267,519 |
| 6 | 'individual':ab,ti OR 'single':ab,ti OR 'conventional':ab,ti OR 'general':ab,ti | 5,057,247 |
| 7 | 'cohort analysis'/exp OR 'longitudinal study'/exp OR 'prospective study'/exp OR 'follow up'/exp OR cohort*:ab,ti | 3,424,942 |
| 8 | #1 OR #2 OR #3 OR #4 | 108,615 |
| 9 | #5 AND #6 AND #7 AND #8 | 762 |
|  | #5 AND #6 AND #7 AND #8 AND [2000-2022]/py | 747 |

**Table S2.** Full search strategy for the Cochrane library (Searched on 03/03/2022).

| **Search number** | **Query** | **Results** |
| --- | --- | --- |
| 1 | MeSH descriptor: [Coronary Artery Bypass] explode all trees | 5603 |
| 2 | (Coronary Artery Bypass Grafting):ti,ab,kw OR (Bypass, Coronary Artery):ti,ab,kw OR (Bypasses, Coronary Artery):ti,ab,kw OR (Artery Bypasses, Coronary):ti,ab,kw OR (Coronary Artery Bypass Surgery):ti,ab,kw OR (Bypasses, Aortocoronary):ti,ab,kw OR (Coronary Artery Bypasses):ti,ab,kw OR (Bypass Surgery, Coronary Artery):ti,ab,kw OR (Bypass, Aortocoronary):ti,ab,kw OR (Artery Bypass, Coronary):ti,ab,kw OR (Aortocoronary Bypass):ti,ab,kw OR (Aortocoronary Bypasses):ti,ab,kw OR (CABG):ti,ab,kw OR (OPCABG):ti,ab,kw | 14,527 |
| 3 | MeSH descriptor: [Coronary Artery Bypass, Off-Pump] explode all trees | 428 |
| 4 | (Coronary Artery Bypass, Off Pump):ti,ab,kw OR (Coronary Artery Bypass, Beating Heart):ti,ab,kw OR (Off-Pump Coronary Artery Bypass):ti,ab,kw OR (Off Pump Coronary Artery Bypass):ti,ab,kw | 1,298 |
| 5 | #1 OR #2 OR #3 OR #4 | 14,538 |
| 6 | (sequential):ti,ab,kw OR (sequence):ti,ab,kw OR (composite):ti,ab,kw OR (snake):ti,ab,kw OR (jump):ti,ab,kw | 65,419 |
| 7 | (individual):ti,ab,kw OR (single):ti,ab,kw OR (conventional):ti,ab,kw OR (general):ti,ab,kw | 402,502 |
| 8 | (cohort analysis):ti,ab,kw OR (longitudinal study):ti,ab,kw OR (prospective study):ti,ab,kw OR (follow up):ti,ab,kw OR (cohort):ti,ab,kw | 462,028 |
| 9 | #5 AND #6 AND #7 AND #8 | 261 |
|  | with Publication Year from 2000to 2022 in Trals |  |

**Table S3.** Full search strategy for PubMed (Searched on 03/03/2022).

| **Search number** | **Query** | **Results** |
| --- | --- | --- |
| 1 | "Coronary Artery Bypass"[Mesh] | 55,266 |
| 2 | (((((((((((((Artery Bypass, Coronary[Title/Abstract]) OR (Artery Bypasses, Coronary[Title/Abstract])) OR (Bypasses, Coronary Artery[Title/Abstract])) OR (Coronary Artery Bypasses[Title/Abstract])) OR (Coronary Artery Bypass Surgery[Title/Abstract])) OR (Bypass, Coronary Artery[Title/Abstract])) OR (Aortocoronary Bypass[Title/Abstract])) OR (Aortocoronary Bypasses[Title/Abstract])) OR (Bypass, Aortocoronary[Title/Abstract])) OR (Bypasses, Aortocoronary[Title/Abstract])) OR (Bypass Surgery, Coronary Artery[Title/Abstract])) OR (Coronary Artery Bypass Grafting[Title/Abstract])) OR (CABG[Title/Abstract])) OR (OPCAB[Title/Abstract]) | 59,388 |
| 3 | "Coronary Artery Bypass, Off-Pump"[Mesh] | 3,541 |
| 4 | ((((Coronary Artery Bypass, Off Pump[Title/Abstract]) OR (Coronary Artery Bypass, Beating Heart[Title/Abstract])) OR (Off-Pump Coronary Artery Bypass[Title/Abstract])) OR (Off Pump Coronary Artery Bypass[Title/Abstract])) OR (Beating Heart Coronary Artery Bypass[Title/Abstract]) | 3,499 |
| 5 | #1 OR #2 OR #3 OR #4 | 76,113 |
| 6 | (sequential[Title/Abstract]) OR (Sequence[Title/Abstract]) OR (composite[Title/Abstract]) OR (snake[Title/Abstract]) OR (jump[Title/Abstract]) | 1,117,970 |
| 7 | (individual[Title/Abstract]) OR (single[Title/Abstract]) OR (conventional[Title/Abstract]) OR (general[Title/Abstract]) | 3,996,023 |
| 8 | cohort studies[mesh:noexp] OR longitudinal studies[mesh:noexp] OR follow-up studies[mesh:noexp] OR prospective studies[mesh:noexp] OR retrospective studies[mesh:noexp] OR cohort[TIAB] OR longitudinal[TIAB] OR prospective[TIAB] OR retrospective[TIAB] OR follow-up[TIAB] | 3,480,243 |
| 9 | #5 AND #6 AND #7 AND #8 | 579 |
| 10 | #5 AND #6 AND #7 AND #8 (Filter: from 2000 - 2022) | 500 |

**Table S4.** Baseline characteristics of included studies.

| **No.** | **First author** | **Published year** | **Setting** | **Sample characteristics** | | | |
| --- | --- | --- | --- | --- | --- | --- | --- |
|  |  |  |  | **Age(AVG ± SMD)** | **Female** | **Other matching features** | **Operation** |
| NO.1 | Zeng | 2021 | China | 58.6±7.3 | 152 | —— | OPCAB |
| NO.2 | Park, S. J. | 2020 | South Korea | （64.6±8.7）/（63.6±9.2） | 257/336 | Diabetes | CABG、OPCAB |
| NO.3 | Wallgren, S. | 2019 | Sweden | 68.7/67.9 | 359/819 | Diabetes、Hypertension、Smoking | —— |
| NO.4 | Skov, J. K. | 2019 | Denmark | （66.5±8.7）/（66.4±8.9） | 622/627 | Diabetes | CABG、OPCAB |
| NO.5 | Takazawa, A. | 2015 | Japan | 71±8 | 126 | —— | CABG、OPCAB |
| NO.6 | Xiao | 2014 | China | （61.3±8.3）/（62.1±9.3） | 27/32 | Diabetes、Hypertension | OPCAB |
| NO.7 | Kim, H. J. | 2011 | South Korea | （62.9±8.3）/（63.7±8.3） | 26/74 | Hypertension | OPCAB、CABG |
| NO.8 | Ouzounian, M. | 2010 | Canada | 66.6±9.7 | 614 | Diabetes、Smoking、Hyperlipidemia、Hypertension | CABG |
| NO.9 | Gao | 2010 | China | 53~82（63.6±10.3） | 97 | —— | OPCAB |
| NO.10 | Silva | 2009 | Brazil | 67±10 | —— | —— | —— |
| NO.11 | Onorati, F. | 2007 | Italy | （76.7±3.9）/（78.69±4.2） | 4/3 | Diabetes、Hypertension、Smoking、Hyperlipidemia | CABG |
| NO.12 | Farsak | 2003 | Turkey | 55.2±9.3 | 66 | Smoking、Diabetes、Hypertension、Blood cholesterol | —— |
| NO.13 | Souza, D. S. | 2002 | Sweden | —— | —— | —— | CABG |
| NO.14 | Vural | 2001 | Turkey | 49±8 | 48 | Atherosclerotic risk factors | —— |
| NO.15 | Dion, R. | 2000 | Belgium | —— | —— | —— | CABG |

Continued

| **Follow-up of study** | | | **Clinical outcomes** | | | | | |
| --- | --- | --- | --- | --- | --- | --- | --- | --- |
| **Sample size** | **Duration (month)** | **Rate** | **Assessment method** | **Occlusion definition** | **Target organ** | **Follow-up time (year)** | **Definition** | **Follow-up time (year)** |
| 311 | 60* | <80% | Angiography | ≥50% | Graft | 5 | —— | —— |
| 2515 | 88(46.3-119.2) | ≥80% | —— | —— | —— | —— | In-hospital mortality, ten-year mortality | 10 |
| 6895 | 35(0-84) | ≥80% | —— | —— | —— | —— | 30-day and 3.5-year mortality, MI, and revascularization | 3.5 |
| 6760 | >12 | ≥80% | —— | —— | —— | —— | In-hospital stroke,and 30-day, 5-year, and 10-year mortality and revascularization | 10 |
| 439 | 14.7±12.5 | —— | CT-angiography/ Angiography | ≥50% | Graft | 1.225 | —— | —— |
| 254 | 21.6 | ≥80% | —— | —— | —— | —— | In-hospital and mid-term mortality, MI, and revascularization | 1.8 |
| 309 | 14.8 | ≥80% | CT-angiography | 100% | Graft | 1.42 | —— | —— |
| 2354 | 78 | ≥80% | —— | —— | —— | —— | In-hospital stroke, mortality, MI, and revascularization | 6.5 |
| 398 | 3~60（19.8±23.6） | —— | CT-angiography | 100% | Graft | 1.65 | —— | —— |
| 88 | 41±25.7 | ≥80% | Angiography | >70% | Graft | 4.41 | —— | —— |
| 90 | 12* | ≥80% | —— | —— | —— | —— | Perioperative mortality, stroke, AMI, early revascularization rate | 1 |
| 509 | 55.4±17.6 | —— | Angiography | 100% | Graft | 4.6 | —— | —— |
| 152 | 16 | ≥80% | Angiography | —— | Graft | 1.33 | —— | —— |
| 430 | 69.6±36 | —— | Angiography | 100% | Graft | 5.8 | —— | —— |
| 500 | 115.2（103.2-163.2） | ＜80% | Angiography | 100% | Graft | 7.5 | —— | —— |

## Supplementary Figures

**Figure S1.** Forest plot of vein graft failure rate in sequential versus individual according to (A) patency evaluation methods, (B) grafting failure definition, (C) different follow-up rates, (D) different surgery methods, and (E) different follow-up time.

**Figure S2.** Forest plot for (A) perioperative mortality and (B) in-hospital stroke.

**Figure S3.** Sensitivity analysis for perioperative mortality (Leave-one-out analysis).

**Figure S4.** Forest plot for (A) mid-term mortality and (B) mid-term repeat revascularization.
